# Association of multidrug resistance behavior of clinical *Pseudomonas aeruginosa* to pigment coloration

**DOI:** 10.1186/s40001-022-00752-6

**Published:** 2022-07-16

**Authors:** Ashish Kothari, Shyam Kishor Kumar, Vanya Singh, Prashant Kumar, Karanvir Kaushal, Atul Pandey, Neeraj Jain, Balram Ji Omar

**Affiliations:** 1 Department of Microbiology, All India Institute of Medical Sciences, Rishikesh, 249203 India; 2 Department of Microbiology, All India Institute of Medical Sciences, Deoghar, 814152 India; 3 Department of Biochemistry, All India Institute of Medical Sciences, Rishikesh, 249203 India; 4 Department of Ecology and Evolutionary Biology, University of Michigan, Ann Arbor, MI 48109 USA; 5 Department of Medical Oncology, All India Institute of Medical Sciences, Rishikesh, 249203 India; 6 Division of Cancer Biology, Central Drug Research Institute, Lucknow, 226031 India

**Keywords:** Pigment production, β-Lactamase, Minimal inhibitory concentration, Biofilm, Drug efflux, Multidrug resistance

## Abstract

*Pseudomonas aeruginosa* is an adaptable bacterial pathogen that infects a variety of organs, including the respiratory tract, vascular system, urinary tract, and central nervous system, causing significant morbidity and mortality. As the primary goal of this study, we wanted to determine how pigment color production differed between clinical strains of *P. aeruginosa*, and whether or not that variation was associated with multidrug resistance or the ability to form biofilms. We screened in total 30.1% of yellow, 39.8% green and 30.1% of no pigment-producing *P. aeruginosa* strains from a total of 143 various clinical isolates. Yellow pigment-producing strains presented significant resistance to antibiotics groups, including β-lactam (91.5%), aminoglycosides (70.5%), and carbapenems (51.9%) compared to green and non-pigmented strains. Notably, 16.3% of yellow pigment-producing strains were resistant to colistin which is used as a last-resort treatment for multidrug-resistant bacteria, whereas only 2.3% of non-pigmented and 1.8% of green pigmented strains were resistant to colistin. Aside from that, yellow pigment-producing strains were frequent producers of enzymes belonging to the lactamase family, including ESBL (55.6%), MBL (55.6%), and AmpC (50%). Compared to the green groups (7.14%) and non-pigmented groups (28.5%), they had a higher frequency of efflux positive groups (64.2%). Notably, when compared to non-pigmented groups, green pigment-producing strains also displayed antibiotic susceptibility behavior similar to yellow pigment-producing strains. The majority of yellow pigment-producing strains outperformed the green and non-pigmented strains in terms of MIC levels when compared to the other two groups of strains. Despite the fact that previous studies have demonstrated a direct correlation between multidrug resistance behaviors and biofilm production, no such statistically significant association between pigment and biofilm formation was found in our investigation. Our research has demonstrated that the correlation of bacterial pigments on their susceptibility to antimicrobial agents. Yellow pigment-producing *P. aeruginosa* strains posed a significant problem due to the lack of alternative agents against such transformed strains, which may be associated with the development of multidrug resistance.

**Graphical Abstract:**

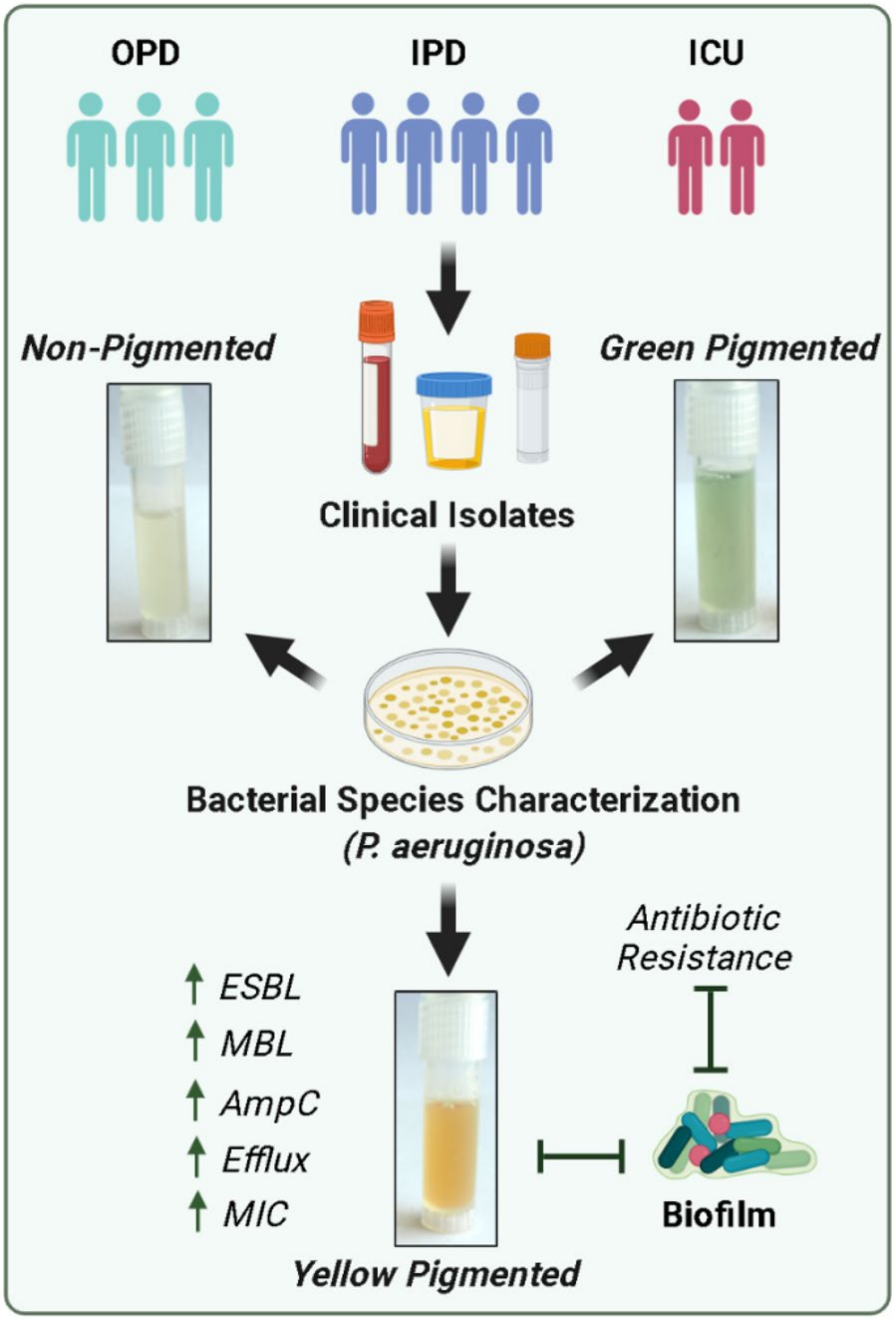

**Supplementary Information:**

The online version contains supplementary material available at 10.1186/s40001-022-00752-6.

## Introduction

*Pseudomonas aeruginosa* strains that are difficult to treat, such as multidrug-resistant (MDR) and extensively drug-resistant (XDR), have risen to become the leading cause of nosocomial infection in humans and pose a life-threatening threat to immunocompromised patients in health care settings [1–4]. In spite of the fact that limiting-dose of antibiotics given in bits may strengthen sensitive bacteria and transformed them into resistant versions, the other imperative factors such as bacterial genomic mutations and acquisition of resistance genes, particularly those encoding extended-spectrum β-lactamases (ESBL) or carbapenemases through horizontal gene transfer, are also attributed to acquired resistance [5–8]. The spread of so-called “high-risk” clones of *P. aeruginosa*, in particular, poses a threat to global public health, necessitating extensive microbiological research and disease management to prevent their spread [8].

The pigments found in every organism, including lower organisms such as microbes and higher organisms, such as plants and animals, serve a function. In microorganisms, pigments can be found in two forms: diffusible/water-soluble pigments that are secreted into the environment, and non-diffusible pigments that are retained within the organism. Most of the *P. aeruginosa* produces one or more extracellular pigments, including pyoverdine (yellow–green and fluorescent), pyocyanin (blue–green), pyorubrin (red–brown), and pyomelanin (brown–black) [9, 10]. These pigments are involved in multiple phenomena, such as quorum sensing network, virulence factor, antioxidant, and iron acquisition properties [11–13].

In recent years, several studies have explored the potential role of pigment production in *P. aeruginosa* pathogenesis. Laura et al. have demonstrated that the contribution of pyomelanin production in *P. aeruginosa* provided resistance to oxidative stress as well as persistent chronic infection properties in a laboratory setting [13]. Notably, in a murine pneumonia model, a strong correlation between pyoverdine production and virulence behavior of a *P. aeruginosa* strain (isolated from cystic fibrosis patients) has been demonstrated, and disruption of pyoverdine production by the specific inhibitor 5-fluorocytosine improved mice survival during infection and mitigated multidrug-resistant pathogenesis [14]. Pigment production appeared to be associated with virulent properties as expressions of virulence-associated genes (exoS, rhlA and rhlB) were more predominant in pigmented isolates than the non-pigmented clinical strains, suggesting that analyzing pigment production in clinical isolates can be a good initiative to determine virulence status of clinical strains [15].

Other important factors such as production of different class of enzymes called “β-lactamases”, which include ESBLs, AmpC β-lactamases and Metallo-β-lactamases (MBL) and non-enzymatic mechanism (efflux- or porin-mediated, outer membrane impermeability, pigment production, and horizontal gene transfer, etc.) are found to be associated with increased virulence and MDR behavior of *P. aeruginosa* isolated from clinical strains [16–19]. Notably, the relationship between these factors (ESBL, MBL, AmpC β-lactamases, efflux pumps, horizontal gene transfer, etc.) and biofilm production, which is another factor in the pathogenesis of *P. aeruginosa*, has also been established [20–23]. However, studies describing the clinical association between pigment production, MDR behavior and biofilm production are minimal [24, 25]. In this study, we have demonstrated the relationship between pigment production status and MDR behavior, biofilm production, β-lactamases producing, and efflux group of *P. aeruginosa* strains isolated from various clinical isolates at the All-India Institute of Medical Sciences, Rishikesh, India.

## Materials and methods

### Ethics statement and clinical samples

This study was approved by the Institutional Ethical Committee of the All-India Institute of Medical Sciences in Rishikesh, India, under the protocol number ECR/736/Inst/UK/2015/RR-18. A total of 143 consecutive samples were selected of those identified to be infected with *P. aeruginosa* strain. From infected patients who were enrolled in respective departments, outside patients (OPD), inside patients (IPD), and the intensive care unit (ICU), samples were collected from a variety of sites, including pus and pleural fluid as well as urine, blood, sputum, and other discharges. Samples after collection were immediately transported to the Microbiology lab and processed immediately as per routine hospital procedure of examination including bacterial identification, gram staining (HIMEDIA, K001), colony morphology (size, shape, texture, opacity), motility (hanging drop method), pigment production (identified by color production), oxidase reaction (Oxidase disc, DD018), and other routine microbiological procedures [26]. Further species identification for the isolates was carried out using Bruker’s MALDI Biotyper Microbial Identification system (Bruker, USA).

### Pigment identification

Color of pigment production was detected by qualitative observation of bacterial growth on preformed King’s A medium agar (HiMedia, M1543). Inoculated bacteria were allowed to grow for 16 h at 37 °C. Colonies that appeared blue–green in texture were considered pyocyanin producers, while–yellow colonies were considered as pyoverdine producers (Additional file 1: Figure S1A).

### Antibiotic susceptibility test (AST)

We have performed a disc diffusion method to test the AST of *P. aeruginosa* strains according to the Kirby–Bauer disk diffusion method on Muller–Hinton agar (MHA) [27]. We have also utilized an automated method for AST detection using a MicroScan WalkAway 96 Plus ID/AST system (Beckman Coulter, Inc., USA) equipped with software suitable for the interpretation of susceptibility testing results as per manufacturer's guidelines. Results of AST were interpreted and categorized as susceptible (S), Intermediate (I) and Resistant (R) according to the Clinical Laboratory Standard Institute (CLSI) guidelines.

### Extended-spectrum β-lactamase detection

ESBL production from all *P. aeruginosa* isolates was detected by Beckman Coulter Microscan Walk-Away and double-disc synergy methods. ESBL production was screened by disc diffusion assay using Ceftazidime (30 μg) and Ceftazidime/Clavulanic acid (30/10 μg) discs and Piperacillin (100 μg) and Piperacillin/Tazobactam (100/10 μg) discs. The zones of inhibition for the Ceftazidime and Piperacillin discs were compared to ceftazidime/clavulanic acid and Piperacillin/Tazobactam discs. An increase in zone diameter in the presence of Tazobactam was confirmed as positive of ESBL production according to CLSI 2021 guideline. ESBL positive *ATCC 27853* was used as a control strain. Bacteria showing resistance to at least three different classes of antibiotics were considered multidrug-resistant [28].

### Minimal inhibitory concentration (MIC) of MBL producing P. aeruginosa

To detect MBL production by *P. aeruginosa* isolates, we performed the RAPIDEC^®^ CARBA NP test (Biomerieux-diagnostic) that is rapid and well adapted to detect Carbapenemase producers as described previously [29]. A positive test for MBL producers corresponded to a color change from red to yellow or orange, light orange, or dark orange. No color change indicated MBL-non producers strains (Additional file 1: Figure S2). MBL positive producers were further utilized for MIC determination for 7 antibiotics (Cefepime, Netilmicin, Gentamicin, Ciprofloxacin, Imipenem, Aztreonam, and Ceftazidime), respectively.

For MIC, we used antimicrobial gradient method Ezy MIC^™^ Strips (HiMedia). In brief, overnight culture of *P. aeruginosa* clinical isolates were diluted in peptone water to a turbidity of 0.5 McFarland standards, followed by inoculum transfer onto MH agar plate. We also utilized micro broth dilution method for MIC determination, where a 96-well micro-plate contained varying concentrations of antibiotics. Saline suspension was prepared to test strain equivalent to 0.5 McFarland standards from a 72 h-old subculture. A 100 μl volume of the suspension was added to each micro-plate with antibiotics incubated at 16 to 48 h at 37 °C. We utilized *ATCC 27853* as reference strain. The MIC was defined as the lowest concentration of a test antibiotic that completely inhibited bacterial growth. MIC result was interpreted using CLSI guidelines (Additional file 1: Table S1).

### AmpC β-lactamases production phenotypic detection

AmpC β-lactamase production was phenotypically detected by the Ezy MICTM Strip (HiMedia, EM081) according to the manufacturer's instructions. The test principle comprises a strip impregnated with a concentration gradient of Cefotetan on one half of the strip and Cefotetan with Cloxacillin on the other half of the strip. MICs of Cefotetan alone and Cefotetan with Cloxacillin were determined as recommended by the manufacturer. Ratios of Cefotetan versus Cefotetan/Cloxacillin of ≥ 8 were considered positive for AmpC beta-lactamase production.

### Efflux pumps detection by ethidium bromide cartwheel method

Trypticase Soy Agar (TSA) (HiMedia, M1969) plates containing ethidium bromide (EtBr) with varying concentrations ranging from 0 to 4 mg/L (these concentrations were determined according to the bacterial MICs for EtBr) were freshly prepared on the same day of the experiment and kept protected from light. Overnight cultures of tested bacterial isolates were adjusted to a 0.5 McFarland turbidity standard. The TSA plates of 9.0-cm diameter was divided into ten to twelve sectors forming a cartwheel (CW) pattern. The adjusted bacterial cultures were swabbed on the EtBr-TSA plates starting from the plate's center to the margin. After incubating the plates at 37 °C for 16 h, the plates were examined under a gel documentation system (Bio-rad, USA). The isolates were considered EtBr-CW-negative if they showed fluorescence emission at 0.5–1 mg/L EtBr, EtBr-CW intermediate (emitting fluorescence at 2.0 mg/L) or EtBr-CW-positive (emitting fluorescence only at 3–4 mg/L). *Staphylococcus aureus ATCC 25923* strain was used as negative control for the efflux experiment.

### Biofilm assay and quantification

Biofilm formation assay was performed as described previously [30]. In brief, overnight culture of *P. aeruginosa* strain was diluted to OD of 0.5 according to McFarland standard. Suspension's culture was further diluted at 1:100 in 200 μL Luria Bertani broth (LB broth) (HiMedia M1245). It was then transferred into the sterile microtiter plate (96-well plates) and incubated in a static culture at 37 °C for 3 days. The culture was discarded after three days and wells were washed with sterile phosphate-buffered saline pH 7.3, and fixed in formaldehyde (10%) for 15 min. The wells were air-dried and stained with crystal violet to stain biofilm (0.1% in ethanol) (HiMedia) for 5 min. Wells were washed with deionized water to remove unbound dye and biofilm bound dye was eluted with ethanol. The optical density (OD) was measured at 550 nm using a plate reader (Eon Microplate Spectrophotometer by BioTek Instruments, Inc) and performed in triplicate. To distribute isolates as strong, moderate, and weak and no-biofilm producing strains, cut off values of OD was set up that represented mean ± standard deviations (SD) of three independent experiments. The respective cutoff value of OD for strong (1.0 ± 0.2), moderate (0.64 ± 0.08), weak (0.12 ± 0.01), and no-biofilm (0.03 ± 0.00) were considered.

### Statistical analysis

All data were analyzed and plotted using GraphPad Prism 5.02 software (GraphPad, La Jolla, CA, USA) and Microsoft excel. The difference between the groups and statistical significance were determined using ANOWA, chi-square test and *T* test, the statistical test of the each investigation is mansion in figure legends. The results are presented as numbers and percentages at a significance level of *p* ≤ 0.05.

## Results

### Prevalence of pigmented and non-pigmented P. aeruginosa clinical strains

**Figure. 1:**
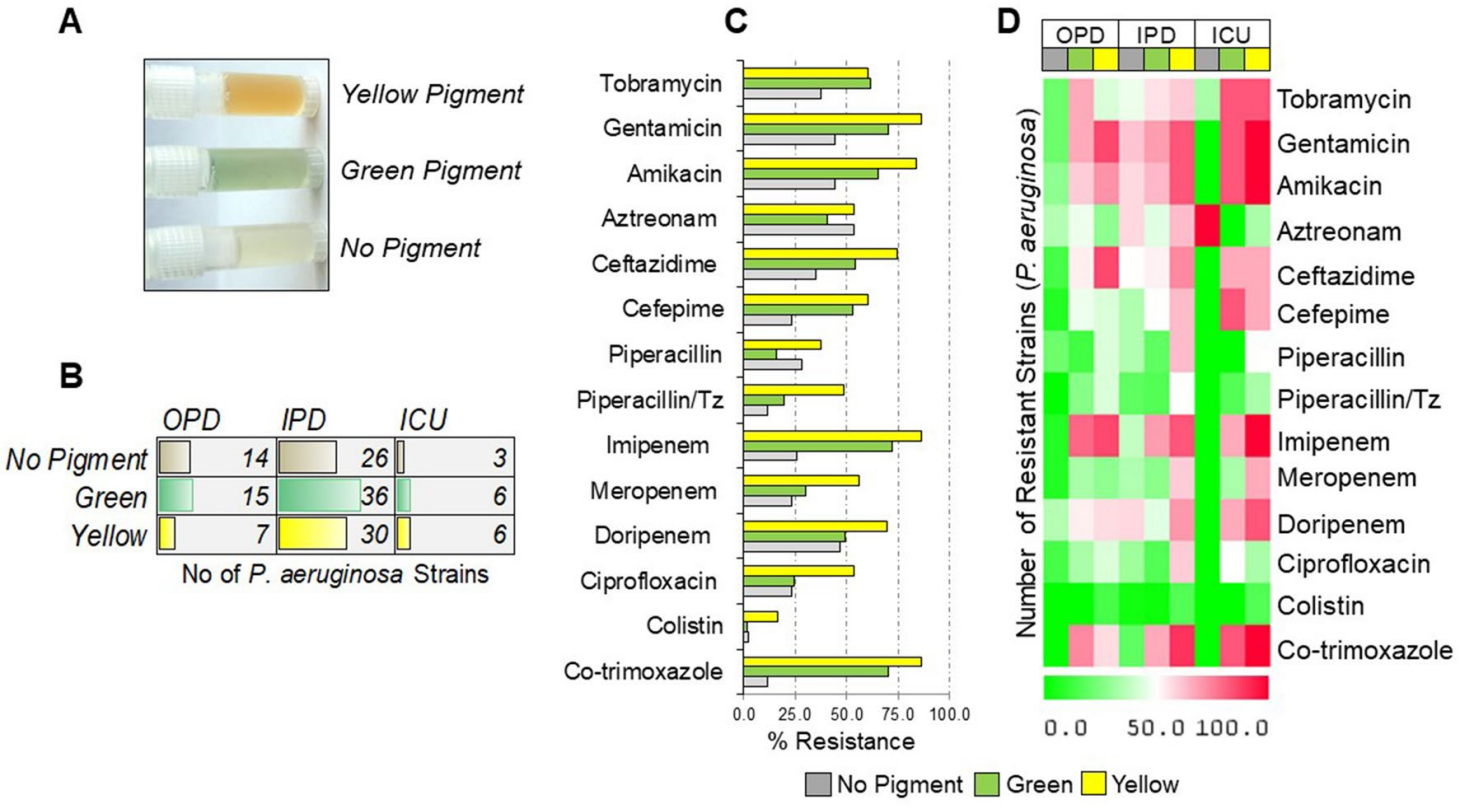
Antibiotic Resistance pattern of clinical *P. aeruginosa* strains: **A** Represented image of pigmented (green and yellow) and non-pigmented clinical strain. **B** Data bar showing numbers of pigmented and non-pigmented strains from different departments. **C** % resistance of green, yellow and no pigment-producing strains with respect to represented antibiotics. **D** Heat-map representing number of respective antibiotic-resistant strains relative to pigmented and non-pigmented group in OPD, IPD and ICU department

A total of 143 clinical isolates were obtained from OPD, IPD and ICU departments that were identified to be infected with *P. aeruginosa*. Among them, 92 (64.5%) were appeared in IPD department. Of the 143 patients, 93 (65.1%) were males and 50 (34.9%) were females (*P* < *0.05*). Determination of mucoid and non-mucoid isolates was investigated phenotypically based on colony appearance and Congo red agar assay. Both non-mucoid 66 (46.1%) and mucoid 77 (53.8%) group of *P. aeruginosa* were identified (*P* > *0.05)* (Table 1). Based on pigment production in Kings medium, 43 (30.1%) of 143 archived isolates produced yellow pigment, whereas 57 (39.8%) were green and 43 (30.1%) with no pigment-producing strains, respectively (*P* > *0.05)* (Figure. 1A). As the number of samples collected from the IPD department was higher, the relative distribution of green, yellow, and no pigment-producing strains was higher in IPD than in the other hospital department (Figure. 1B).

**Table 1:** Demographic and Clinical Characteristics of *P. aeruginosa* infected patients from OPD, IPD, and ICU

Characteristics	OPD n {%} 36{25.1}	IPD n {%} n = 92{64.5}	ICU n {%} n = 15{10.4}	Total n {%} n = 143
Age				
Gender				
Male	23{63.8}	58{63}	12{80}	93{65.1}
Female	13{36.1}	34{36.9}	3{20}	50{34.9}
Pigment status				
No Pigment	14{38.8}	26{28.2}	3{20}	43{30}
Green	15{26.3}	36{39.1}	6{40}	57{39.8}
Yellow	7{19.4}	30{32.6}	6{40}	43{30}
Mucus status				
Non-mucoid	20{55.5}	38{41.3}	7{46.6}	66{46.1}
Mucoid	16{44.4}	54{58.6}	8{53.3}	77{53.8}

### Antimicrobial resistance pattern of pigmented and non-pigmented P. aeruginosa clinical strains

We next evaluated the antibiotic susceptibility pattern using a group of 14 anti-pseudomonal drugs. Pigmented strains (green and yellow producing strains) showed more resistance to tested antibiotics than non-pigmented ones (Figure. 1C). Among the pigmented and non-pigmented groups, the yellow pigment-producing strains were more resistant to most drugs tested than green and no pigment-producing strains (Figure. 1C and Additional file 1: Figure S1B, C). The resistance frequency (in percentage) of non-pigmented, green, and yellow strains for represented antibiotics were: Colistin (2.3, 1.8, 16.3), Ciprofloxacin (23.3, 24.6, 53.5), Doripenem (46.5, 49.1, 69.8), Meropenem (23.3, 29.8, 55.8), Piperacillin/Tz (11.6, 19.3, 48.8), Piperacillin (27.9, 15.8, 37.2), Ceftazidime (34.9, 54.4, 74.4), and Aztreonam (53.5, 40.4, 53.5), respectively (Figure. 1C). Similar results were obtained when patients were grouped into OPD, IPD, and ICU departments, where yellow pigment-producing strains presented more frequent resistant patterns than green and non-pigmented strains (Figure. 1D). Notably, patients from ICU showed a profound resistance pattern in yellow pigment-producing strains than IPD and OPD patients (Figure. 1D and Table 2).

**Table 2:** Resistance pattern of *P. aeruginosa* strains isolated from different departments OPD, IPD, and ICU for listed classes of antibiotics

Groups of Antibiotics	Antibiotics	Non-pigment			Green			yellow			Total
		OPD {n = 14}	IPD {n = 26}	ICU {n = 3}	OPD {n = 15}	IPD {n = 36}	ICU {n = 6}	OPD {n = 7}	IPD {n = 30}	ICU {n = 6}	143
Aminoglycosides	Tobramycin	3{21.4}	12{46.1}	1{33.3}	10{66.6}	20{55.5}	5{83.3}	3{42.8}	18{60}	5{83.3}	77{53.8}
	Gentamicin	3{21.4}	16{61.5}	0{0}	10{66.6}	25{69.4}	5{83.3}	6{85.7}	25{83.3}	6{100}	96{67.1}
	Amikacin	4{28.5}	15{57.6}	0{0}	9{60}	23{63.8}	5{83.3}	5{71.4}	25{83.3}	6{100}	92{64.3}
Β-Lactams	Aztreonam	5{35.7}	15{57.6}	3{100}	7{46.6}	16{44.4}	0{0}	2{28.5}	19{63.3}	2{33.3}	69{48.2}
	Ceftazidime	2{14.2}	13{50}	0{0}	8{53.3}	19{52.7}	4{66.6}	6{85.7}	22{73.3}	4{66.6}	78{54.5}
	Cefepime	1{7.1}	9{34.6}	0{0}	7{46.6}	18{50}	5{83.3}	3{42.8}	19{63.3}	4{66.6}	66{46.1}
	Piperacillin	3{21.4}	9{34.6}	0{0}	2{13.3}	7{19.4}	0{0}	3{42.8}	19{63.3}	3{50}	46{32.1}
	Piperacillin/Tz	0{0}	5{19.2}	0{0}	4{26.6}	6{16.6}	1{16.6}	3{42.8}	15{50}	3{33.3}	36{25.1}
Carbapenem	Imipenem	1{7.1}	10{38.4}	0{0}	12{80}	25{69.4}	4{66.6}	6{85.7}	25{83.3}	6{100}	89{62.2}
	Meropenem	1{7.1}	9{34.6}	0{0}	5{33.3}	10{27.7}	2{33.3}	2{28.5}	18{60}	4{66.6}	51{35.6}
	Doripenem	5{35.7}	15{57.6}	0{0}	8{53.3}	16{44.4}	4{66.6}	4{57.1}	21{70}	5{83.3}	78{45.5}
Fluoroquinolones	Ciprofloxacin	2{14.2}	8{30.7}	0{0}	5{33.3}	6{16.6}	3{50}	3{42.8}	18{60}	2{33.3}	47{32.8}
Polymyxin	Colistin	0{0}	1{3.8}	0{0}	0{0}	1{2.7}	0{0}	1{14.2}	5{16.6}	1{16.6}	9{6.2}
Sulfonamides	Co-trimoxazole	0{0}	5{19.2}	0{0}	11{73.3}}	24{66.6}	5{83.3}	4{57.1}	27{90}	6{100}	82{57.3}

**Figure. 2:**
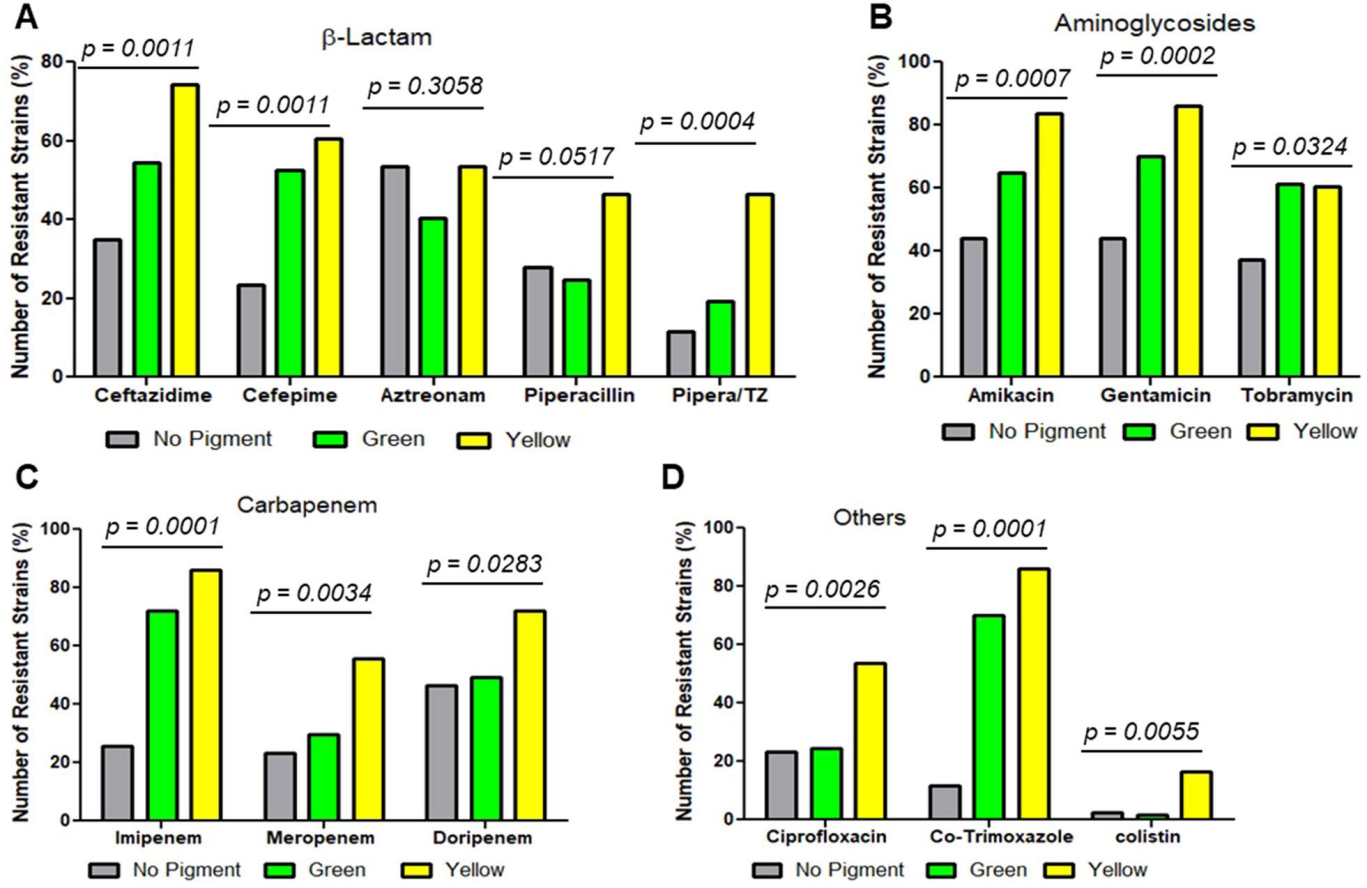
Antibiotic susceptibility pattern according to different classes: **A** *P. aeruginosa* clinical strains representing antibiotic susceptibility to different class of antibiotics. Resistance pattern of green, yellow and no pigment-producing strains among antimicrobial groups of (**B**) aminoglycosides, (**C**) β-lactam, (**D**) carbapenem, (**E**) others including fluoroquinolones (Ciprofloxacin), sulfonamides (Co-trimoxazole), polymyxin (Colistin). *P* < *0.05* is considered statistically significant

On distribution of antibiotics tested into different classes, including aminoglycosides carbapenems, β-lactam and others (fluoroquinolones, Polymyxin, sulfonamides), we found majority of *P. aeruginosa* strains were resistant to β-lactam class of antibiotics (Table 2). Based on pigment production, pigmented strains were more frequently resistant than non-pigmented strains. We found yellow-pigmented strains were strongly and significantly resistant to all classes of antibiotics, followed by green and non-pigmented strains (Figure. 2A–D). In β-lactam group, the prevalence of resistance in yellow strains (91.5%) was highest, followed by in green and non-pigmented strains. Similarly, in aminoglycosides class of antibiotics, the prevalence of resistance was higher as compared carbapenems, and other class of antibiotics for yellow, green and non-pigmented strains, respectively.

### ESBL, MBL, AmpC and efflux pump activity are prevalent in pigmented P. aeruginosa clinical strains

**Figure. 3:**
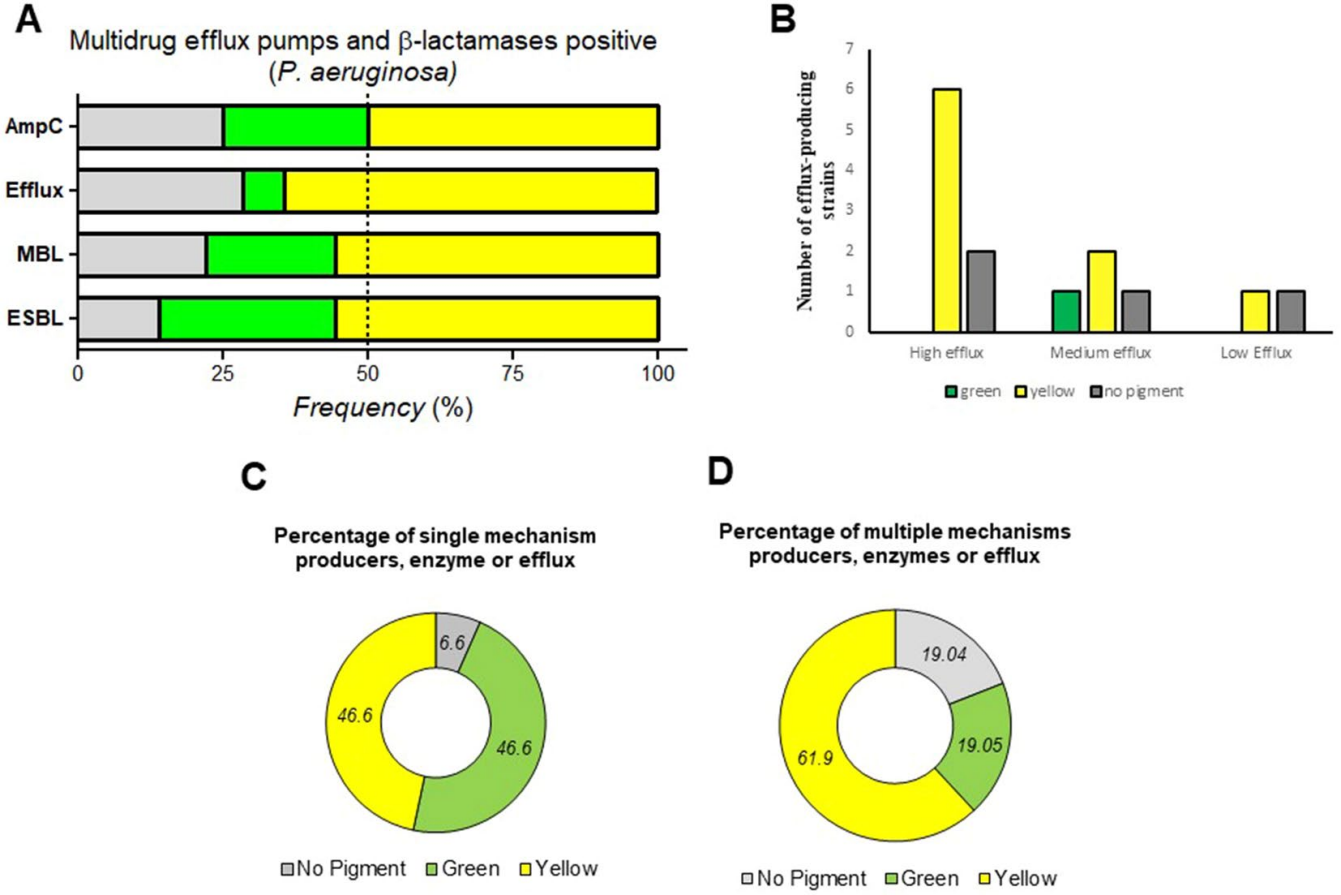
Distribution of antibiotic susceptibility pattern according to efflux and β-lactamases enzyme-producing pigmented and non-pigmented *P. aeruginosa* clinical strains: **A** Frequency of Extended-Spectrum, Metallo-β-Lactamase, AmpC-β-Lactamase and efflux positive strains in pigmented (green and yellow) and non-pigmented groups. **B** Number of high, moderate and low efflux producing green, yellow and non-pigmented strains. A number of yellow, green and non-pigmented strains shown in percentage comprises single (**C**) or combinatorial β-lactamases producing strains

Our distribution analysis based on ESBL, MBL and AmpC producing strains showed yellow-pigmented *P. aeruginosa* were more frequent producers of ESBL (55.6%), MBL (55.6%), and AmpC (50%) then green (ESBL, 30.5%; MBL, 22.2%; AmpC, 25%) and non-pigmented (ESBL, 13.9%; MBL, 22.2%; AmpC, 25%) strains (Figure. 3A and Additional file 1: Figure S2).

We next evaluated efflux activity of isolates by a fluorometric assay that detects EtBr efflux and presents the ability to pump EtBr out of the cell. We selected all *P. aeruginosa* clinical isolates that presented resistance towards Ciprofloxacin (47 out of 143) by automated methods (Microscan Walk-Away 96 plus) and manual disc diffusion methods. Out of 47 Ciprofloxacin-resistant strains, 14 strains (29.8%) did not retain EtBr at various levels when tested on agar plates containing EtBr (Additional file 1: Figure S3). Among 14 positive efflux strains, 9 of them were yellow pigment producers, 1 was green pigment producer, and 4 were non-pigmented strains (Figure. 3A). Further characterization of efflux positive strain based on high, moderate and low efflux activity showed majority of yellow pigment producers had high efflux activity (Figure. 3B).

We further determine the frequency of one single mechanism producers (efflux- or enzyme-positive) and that of co-producers (positive for two or more than two enzymes, or enzyme plus efflux). We found, the yellow and green pigment-producing strains were equally distributed (46.6%) among single enzyme/efflux positive producers, whereas non-pigmented were less frequent (6.6%) (Figure. 3C). Where, yellow pigment-producing strains were highly frequent (61.9%), then green (19.04%) and non-pigmented (19.04%) strains among co-producers strains (Figure. 3D).

### MIC of MBL producing pigmented and non-pigmented strains

**Figure. 4:**
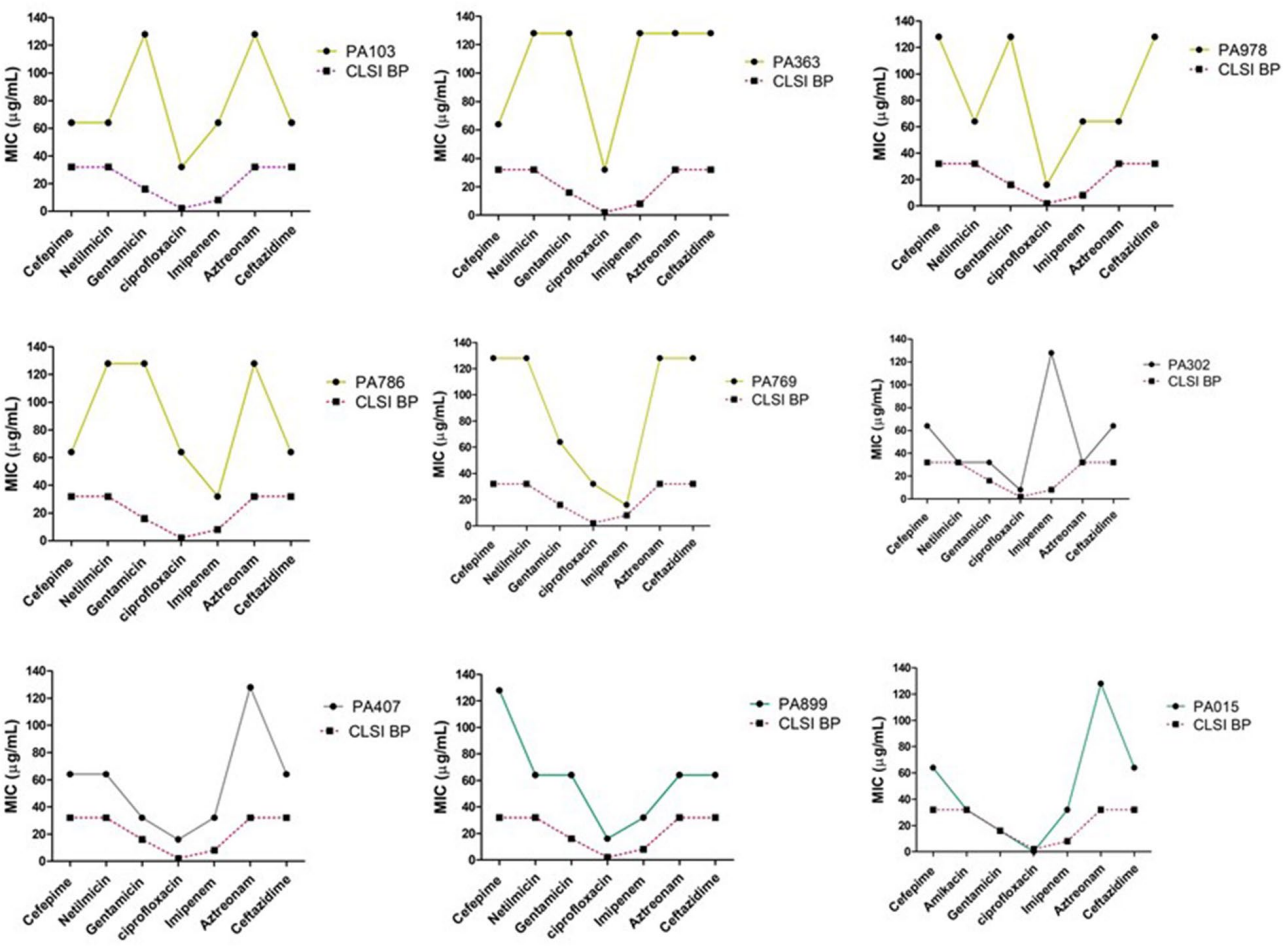
MIC of Metallo-beta-lactamase-producing pigmented and non-pigmented *P. aeruginosa*. Comparative MIC pattern for tested antibiotics according to Clinical & Laboratory Standards Institute guidelines for yellow pigment (PA103, PA363, PA978, PA786, PA769), (**A**–**E**); non pigmented (PA302, PA407), (**F**, **G**); and green pigmented producing strains (PA899, PA015) (**H**, **I**)

MBL producing *P. aeruginosa* strains threaten individuals and are associated with higher morbidity mortality rates, especially in immunocompromised patients [31]. We have screened the MIC level of all MBL producing clinical strains (PA103, PA363, PA978, PA786, PA769, PA302, PA407, PA899, and PA015). Compared to reference strain *ATCC 27853*, all MBL positive clinical strains have shown MIC levels greater than the MIC breakpoints recommended by CLSI guidelines with all 7 individual antibiotics tested (except the strain PA015 showed sensitivity to ciprofloxacin) (Additional file 1: Table S1). Yellow pigment-producing strains have shown MIC levels even greater than green and non-pigmented strains (Figure. 4A–I).

### Biofilm production of pigmented and non-pigmented strains

**Figure. 5:**
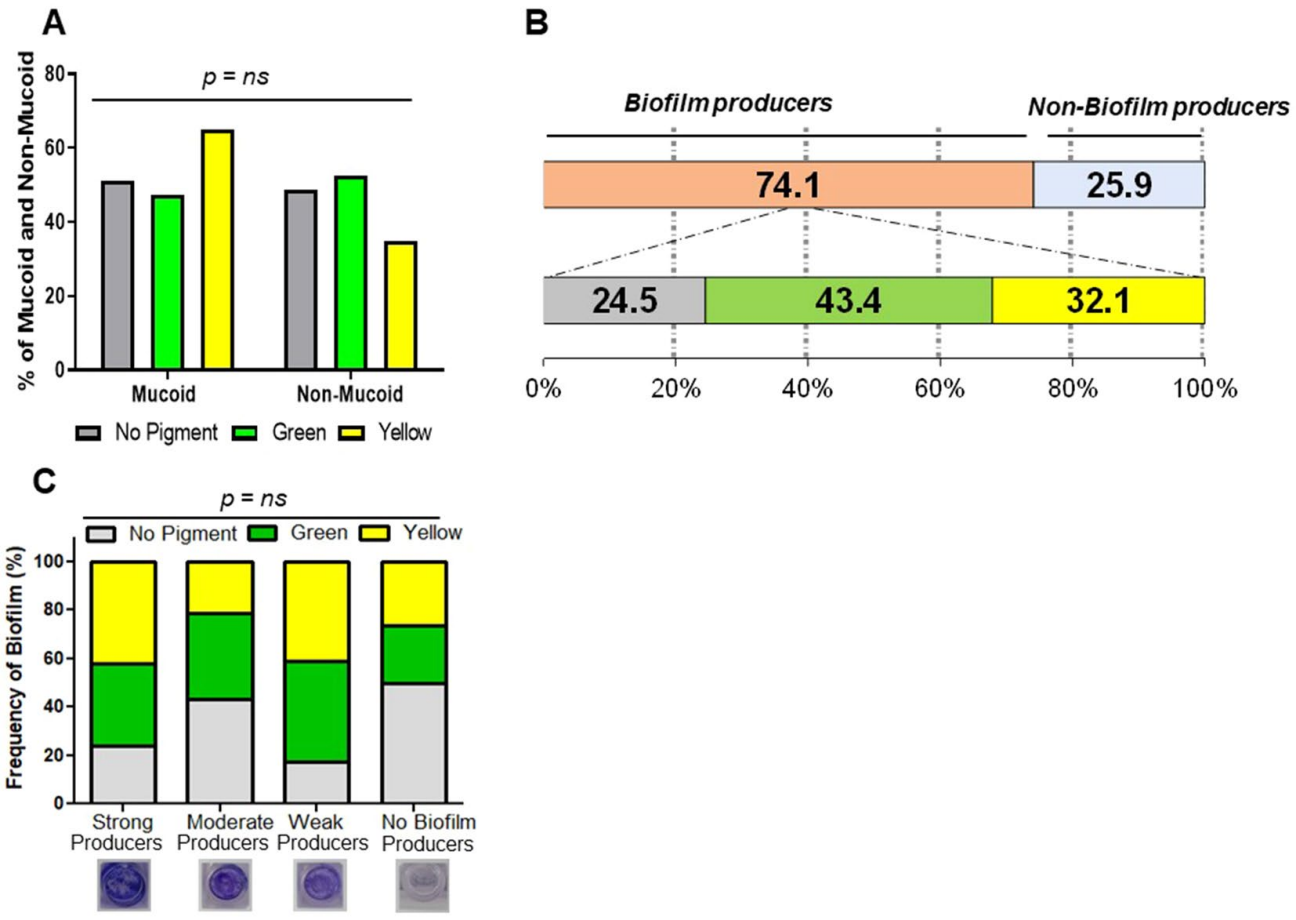
Biofilm producing property of pigmented and non-pigmented *P. aeruginosa* strains: (**A**) relative percentage of pigmented and non-pigmented strains confined mucoid or non-mucoid phenotype. **B** Percentage of biofilm-producing and no biofilm-producing pigmented and non-pigmented strains. **C** Frequency of strong, moderate, and weak biofilm producers among no pigmented and pigmented strains, ns = not significant

Mucoid and non-mucoid colonies are associated with biofilm production and resistance to antibiotics, respectively. We do not observe a significant difference in biofilm formation between mucoid and non-mucoid strains (Additional file 1: Figure S4A). However, mucoid strains were frequently resistant to Piperacillin/Tz, Piperacillin, Meropenem, Aztreonam and Doripenem then non-mucoid strains (Additional file 1: Figure S4B). Among the mucoid and non-mucoid groups, there was no relative difference observed in the frequency of yellow, green and non-pigmented strains (Figure. 5A). To test the biofilm formation ability of clinical strains, we screened all 143 yellow, green and non-pigmented *P. aeruginosa* strains. We did not find a correlation of antibiotic resistance patterns between biofilm and non-biofilm producers (Additional file 1: Figure S4C). Out of 143 strains, 106 (74.1%) were biofilm producers, in which 34 (32.1%) were yellow, 46 (43.4%) green and 26 (24.5%) non pigmented strains produced biofilm (Figure. 5B). Based on OD received on microtiter plate, we have further categorized biofilm producers as strong, moderate and weak biofilm producers. Comparative analysis among yellow, green and non-pigmented strains showed 16 (37.2%) of yellow, 17 (29.8%) of green and 9 (20.9%) of non-pigmented were strong biofilm producers. Whereas 6 (13.9%) of yellow, 13 (22.8%) of green and 12 (27.9%) of non-pigmented were moderate biofilm producers; and 6 (27.9%) of yellow, 13 (28.1%) of green and 12 (11.6%) of non-pigmented were weak biofilm producers (Figure. 5C).

## Discussion

The increasing prevalence of multidrug-resistant bacterial strains, particularly for the most common clinical pathogen *P. aeruginosa* in human isolates, presents a significant challenge in the identification of new treatment strategies, which can result in high rates of morbidity and mortality in hospitals and other healthcare facilities. Fortunately, a number of rapid methods have been developed to identify such MDR species and antibiotic susceptibility within a couple of hours from clinical isolates that have benefited from the reduction in increasing mortality [32–35]. Common characteristics for *P. aeruginosa* to represent the MDR behavior in clinical strains are the presence of drug resistance genes or its mutant variety in bacterial plasmid or in its genome, high expression of a β-lactamase group of enzymes, and up-regulation of drug efflux pump have been documented [36–39]. Interestingly, a recent effort using proteomic profiling has characterized antibiotics sensitive and MDR clinical strains of *P. aeruginosa* that provided non-genetic changes associated with antibiotic susceptibility responses [40].

Pigment production such as pyocyanin and pyoverdin are important virulent factors that augment bacterial virulence via diverse mechanisms [13–15]. To the best of our knowledge, the characterization of MDR signatures of *P. aeruginosa* from clinical strains on the basis of pigment production is extremely limited [13–15]. This study presents a strong association of pigment coloration produced by *P. aeruginosa* strains from clinical isolates with MDR behavior, efflux activity, and biofilm formation. First and foremost, we notified three major groups of *P. aeruginosa*: green, yellow, and no pigment-producing strains from our cohort of clinical samples. It should be noted that we have also identified intermittent red and brown pigment-producing strains, but we did not have enough samples to conduct an analysis. As a result, they have been excluded from this study. Among the pigmented and non-pigmented strains, the yellow pigment producing strain demonstrated profound resistance behavior with the majority of antibiotics tested in clinical samples archived from IPD and ICU departments, indicating that this yellow pigment producing strain may be a serious risk factor. A remarkable resistance pattern of yellow-pigmented strain was noted for different antibiotic groups, including aminoglycosides, fluoroquinolones, carbapenem, sulfonamides, Polymyxin and β-lactams.

Acquired resistance by producing ESBL, MBL and AmpC enzymes is a common phenomenon *of P. aeruginosa* [41]. Phenotypic methods applied in this study helped in detecting *P. aeruginosa* isolates producing various ESBL, MBL, AmpC, enzymes, and efflux activity against different antibiotics. Despite the fact that the frequency of these enzyme-producing strains in our cohort of 143 clinical isolates was not higher than that found in other studies, the prevalence of these enzyme-producing organisms could vary depending on geographic origins, infection patterns, hospital infection control measures, and different departments within the same hospital, among other factors[16, 35]. To our particular surprise, our yellow pigment-producing strains were more frequently found to be enzyme producers and to have co-occurrences with more than one enzyme/efflux positivity than either our green or non-pigmented strains. Furthermore, the identification of MIC for MBL producing strains revealed a higher MIC level of yellow pigment producing strain than the CSLI MIC breakpoints, indicating that the MIC level of yellow pigment producing strain was increased.

The formation of biofilms is an important mechanism. The survival of *P. aeruginosa* causes significant problems, and the structures that the bacteria develop because increased resistance to antibiotic treatment. There has been some evidence of an inverse relationship between biofilm formation and the expression of MDR genes in a few research studies [42, 43]. In our study, though a significant number (74.13%) of isolates formed biofilm, we did not observe a strong correlation of biofilm formation with pigment production.

In conclusion, green pigmented strains exhibited a moderate resistance pattern when compared to yellow and non-pigmented strains, indicating that pigment-producing strains may be more associated with resistance to antimicrobial agents than non-pigmented strains and that comprehensive testing for antibiotic susceptibility pattern should be performed prior to making a treatment recommendation to avoid detrimental effects.

## Conclusions

For the first time, we have demonstrated a relationship between pigment coloration and MDR behavior in *P. aeruginosa* isolates obtained from a variety of clinical samples in our present study. Furthermore, this study demonstrated that the different pigmented *P. aeruginosa* isolates were resistant to ESBL, MBL, AmpC, and efflux mediated resistance. Despite the fact that our study, which included multiple experiments, revealed an association between pigment coloration and antibiotic resistance behavior, the limitations of our study is lack of molecular investigations. *P. aeruginosa* strains that produce yellow pigment color were found to have resistance patterns to more than one type of antibiotic group, the researchers discovered. As a result, determining the most appropriate antibiotic for treatment is critical, as the misuse or overuse of antibiotics can result in a significant increase in the risk of the emergence of antibiotic resistance. This may be a good starting point for determining the multi-drug resistance status of an isolate, because pigment production can be easily determined in most bacteria. For this observation to be confirmed, however, additional research must be conducted.

## Supplementary Information

**Additional file 1: Figure S1.** Pigment production on King A medium agar plate and antibiotic susceptibility behavior. (A) *P. aeruginosa* clinical strain on agar plates showing yellow (a) and green (b) coloration. Disc diffusion on Mueller Hinton agar displayed antibiotic susceptibility of green (B) and yellow (C) pigment producing clinical strains. **Figure S2.** Representative images of Rapidec Carba NP test for MBL producing *P. aeruginosa* clinical strain. The reaction's positivity must be read in well ‘e’, while well ‘d’ is a control that must be red to validate the test. Yellow color in well “e” represents strong positive result for MBL producers (indicated by solid arrow). Weak MBL producers are represented by orange, light orange, dark orange color in obtained in well “e” (indicated by dotted arrow). Red color in well “e” indicates MBL negative isolate. We identified yellow pigment producing strains were primarily strong MBL producers compared followed by green and no-pigment producing strains.

## Abbreviations

AmpC: Ampicillin-hydrolyzing cephalosporinase
ESBL: Extended spectrum β-lactamase
MBL: Metallo-β-lactamase
EtBr-CW: Ethidium bromide cartwheel
