# Supplementary material for: Association of multidrug resistance behavior of clinical Pseudomonas aeruginosa to pigment coloration
Source: Eur J Med Res. 2022 Jul 16;27:120. doi: 10.1186/s40001-022-00752-6 (PMC9288039; doi:10.1186/s40001-022-00752-6)
Supplement: Supplementary file 1 — Additional file 1: Figure S1. Pigment production on King A medium agar plate and antibiotic susceptibility behavior. (A) P. aeruginosa clinical strain on agar plates showing yellow (a) and green (b) coloration. Disc diffusion on Mueller Hinton agar displayed antibiotic susceptibility of green (B) and yellow (C) pigment producing clinical strains. Figure S2. Representative images of Rapidec Carba NP test for MBL producing P. aeruginosa clinical strain. The reaction's positivity must be read in well ‘e’, while well ‘d’ is a control that must be red to validate the test. Yellow color in well “e” represents strong positive result for MBL producers (indicated by solid arrow). Weak MBL producers are represented by orange, light orange, dark orange color in obtained in well “e” (indicated by dotted arrow). Red color in well “e” indicates MBL negative isolate. We identified yellow pigment producing strains were primarily strong MBL producers compared followed by green and no-pigment producing strains. [file 40001_2022_752_MOESM1_ESM.docx]

**Association of multidrug resistance behavior of clinical *Pseudomonas aeruginosa* to pigment coloration**

Ashish Kothari^1^, Shyam Kishor^2^, Vanya Singh^1^, Prashant Kumar^3^, Karanvir Kaushal^3^, Atul Pandey^4^, Neeraj Jain*^5,6^ and Balram Ji Omar*^1^

^1^Department of Microbiology, All India Institute of Medical Sciences, Rishikesh 249203, India.

^2^Department of Microbiology, All India Institute of Medical Sciences, Deoghar 814152, India.

^3^Department of Biochemistry, All India Institute of Medical Sciences, Rishikesh 249203, India.

^4^Department of Ecology and Evolutionary Biology, University of Michigan, Ann Arbor, Michigan 48109, USA. ^5^Department of Medical Oncology, All India Institute of Medical Sciences, Rishikesh 249203.

^6^Division of Cancer Biology, Central Drug Research Institute, Lucknow 226031.

**Additional file Figures**

**Figure S1: Pigment production on King A medium agar plate and antibiotic susceptibility behavior.** (A) *P. aeruginosa* clinical strain on agar plates showing yellow (a) and green (b) coloration. Disc diffusion on Mueller Hinton agar displayed antibiotic susceptibility of green (B) and yellow (C) pigment producing clinical strains.

**
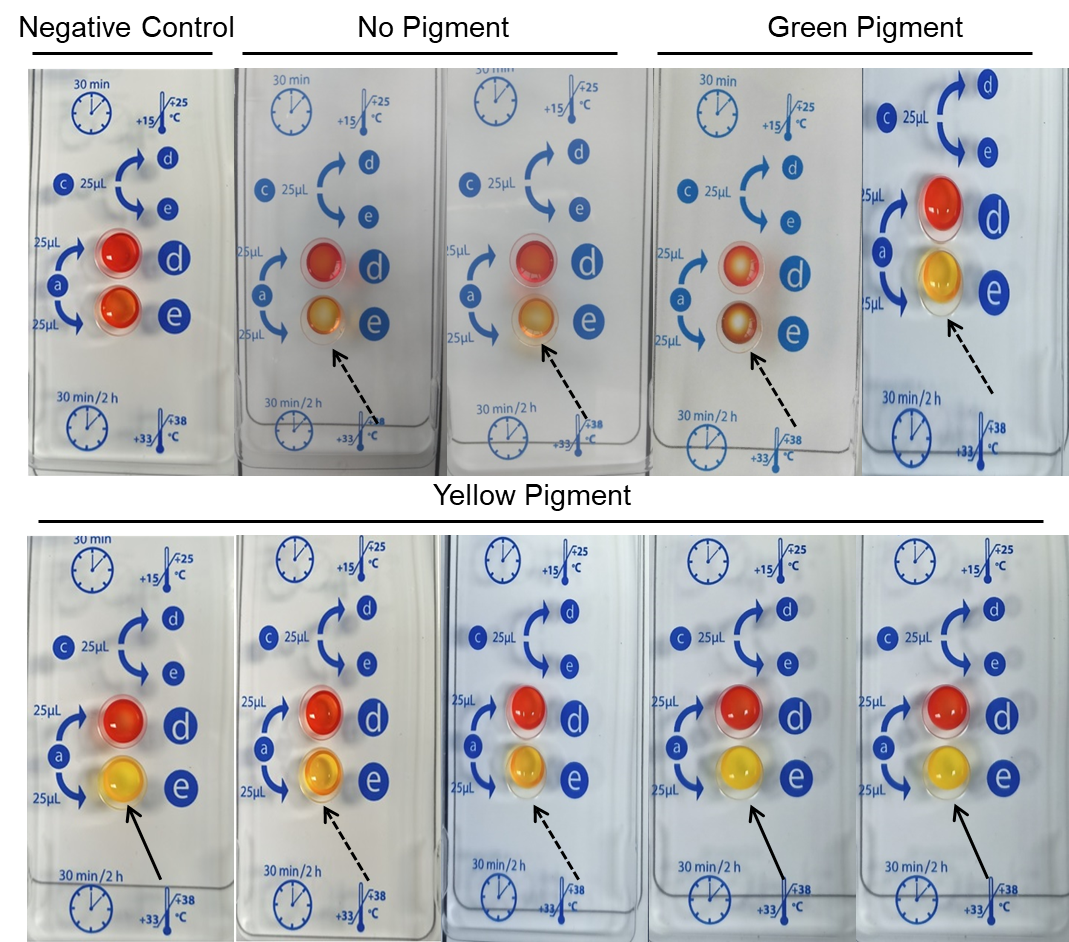
**

**Figure S2: Representative images of Rapidec Carba NP test for MBL producing *P. aeruginosa* clinical strain.** The reaction's positivity must be read in well ‘e’ while well ‘d’ is a control that must be red to validate the test. Yellow color in well “e” represents strong positive result for MBL producers (indicated by solid arrow). Weak MBL producers are represented by orange, light orange, dark orange color in obtained in well “e” (indicated by dotted arrow). Red color in well “e” indicates MBL negative isolate. We identified yellow pigment producing strains were primarily strong MBL producers compared followed by green and no-pigment producing strains.

**Supplementary Figure 3: EtBr Cartwheel assay for drug efflux activity determination.** Fluorescence of *P. aeruginosa* strains on agar plates containing EtBr (2mg/L). Image showing yellow pigment producing strain have enhanced efflux activity (reduced fluorescence) where non-pigmented strain have low efflux activity (high fluorescence). *ATCC 25923* strain was used as negative control for efflux activity.

**Supplementary Figure 4: Mucoid and non-mucoid behavior for biofilm and antibiotic susceptibility. (**A) Biofilm forming mucoid and non-mucoid *P. aeruginosa* clinical strain (shown in %). (B) Difference in resistance pattern (%) for mucoid and non-mucoid strains. (C) Difference in resistance pattern (%) for biofilm producers and non-biofilm producers. # Difference of more than 15% of resistance between groups.
